# Circulating Tumour DNA for Ovarian Cancer Diagnosis and Treatment Monitoring: What Perspectives for Clinical Use?

**DOI:** 10.3390/ijms26051889

**Published:** 2025-02-22

**Authors:** Du-Bois Asante, Domenico Tierno, Gabriele Grassi, Bruna Scaggiante

**Affiliations:** 1 Department of Biomedical and Forensic Sciences, University of Cape Coast, Cape Coast P.O. Box CCLN 33, Ghana; duasante@ucc.edu.gh; 2 Department of Medicine, Surgery and Health Sciences, University of Trieste, Strada di Fiume 447, I-34149 Trieste, Italy; domenico.tierno@units.it (D.T.); ggrassi@units.it (G.G.); 3 Department of Life Sciences, University of Trieste, Via Valerio 28, I-34127 Trieste, Italy

**Keywords:** ovarian cancer, circulating tumour DNA (ctDNA), liquid biopsy, diagnosis, prognosis

## Abstract

Globally, ovarian cancer (OC) is the eighth most common malignant tumour in women. Unfortunately, its symptoms—especially at the early stages—are vague and non-specific, and, thus, most patients are diagnosed at the advanced stages of the disease (stage III and IV) when treatment is not curative. The currently available approved biomarkers are not sufficient for effective screening, prognosis, or monitoring of OC. Liquid biopsy tests such as circulating tumour DNA (ctDNA) analysis has the advantage of monitoring response to treatment in real time and providing a comprehensive genotypic profile of primary, metastatic, and recurrent tumours. Thus, ctDNA analysis can be used as a complementary test for effective diagnosis and monitoring of OC. We comprehensively review current studies (2019–2024) on OC, critically highlighting recent developments and applications of ctDNA for the diagnosis and management of the disease.

## 1. Introduction

Ovarian cancer (OC) is a heterogeneous group of malignancies originating from the ovary and/or fallopian tube [1]. Worldwide, OC is the eighth most common cancer in women and the leading cause of death from gynaecological cancers [2,3], making it one of the deadliest cancers in females. Clinically, the disease only becomes apparent in advanced stages (III and IV) when it is difficult to treat [4]. Given the high mortality rate in advanced stages of OC, early diagnosis remains the most important prognostic factor [2].

The most frequently used screening tests in the clinic are blood analysis for elevated CA-125 protein levels and transvaginal ultrasound (TVUS). Conventional TVUS cannot reliably differentiate between benign and malignant tumours; therefore, invasive surgery may be required for confirmation [5]. On the other hand, serum CA-125 is not elevated in all early stages or in some cases of recurrent OC [6] and is therefore not reliable. In addition, blood tests to determine CA-125 protein levels are not specific for OC, as CA-125 levels can be elevated by various normal physiological and pathological factors [7]. More importantly, CA-125 and TVUS results do not provide direct information about the molecular characteristics of the tumour. OC treatment in advanced stages usually requires a combination of invasive surgery and chemotherapy [8]. The collection of peripheral blood is minimally invasive and can be used as a source of tumour-derived material [9]. Therefore, the introduction of additional and complementary biomarkers with a minimally invasive approach for diagnosis, prognosis, and longitudinal disease monitoring could help improve the survival outcomes of advanced-stage OC patients.

Liquid biopsy tests such as the analysis of circulating tumour DNA (ctDNA) and cells (CTCs) have the advantage of monitoring the response to treatment in real time and providing a comprehensive genotypic profile of primary, metastatic, and recurrent tumours [9]. Thus, the analysis of these biomarkers has the potential to be used as a complementary test for effective diagnosis and monitoring of OC. Herein, we comprehensively review current studies on OC, highlighting recent developments and applications of ctDNA for the diagnosis and management of the disease. We further discuss important caveats that could be considered to enhance improvements in the disease diagnosis and prognosis for favourable patient outcomes.

## 2. CtDNA in Ovarian Cancer

CtDNA is part of the cell-free DNA (cfDNA) that enters the bloodstream in cancer patients. CtDNA carries genetic aberrations that can be identified in matching tumours of an individual. The ctDNA concentrations in cancer patients have been shown to be significantly higher than in healthy individuals [10]. Significant progress has been made in the field of ctDNA analysis in various cancers, including OC [11,12]. In recent years, progress have been made in this field by developing new techniques with high sensitivity and specificity that can comprehensively interrogate a large number of cancer-related genes. CtDNA analysis can also be used to assess DNA alterations such as hypermethylation status in promoter regions, transcriptomics, fragment size patterns, and copy number events and fusions, and this type of analysis enables the assessment of the suitability of the biomarker for the management of cancer patients [12].

We carried out a search of the literature in NCBI PubMed and Scopus from January 2019 to November 2024 (5-year period) using ‘ovarian cancer’ or ‘ovarian carcinoma’ together with ‘circulating tumour DNA’. A total of 45 (35 direct analyses of mutation copies and 10 epigenetic modifications extracted from cell-free DNA) published articles describing the analysis of ctDNA for diagnosis, prognosis, and/or monitoring in OC patients were identified. The inclusion criteria were ctDNA analyses only in OC patients and articles published in the period 2019–2024. Recent ctDNA analyses with participants with gynaecological cancers, including OC, and other types of cancers were excluded. Although most of the studies have focussed on the mutational burden of ctDNA, a considerable number of studies have also been conducted on the epigenetic aspect of the disease by investigating the methylation profile of ctDNA.

Table 1 and Table 2 provide a summary of the studies and the main clinical findings with ctDNA-based mutations analysis (Table 1) and with ctDNA methylation analysis (Table 2).

### 2.1. Diagnostic and Predictive Potential of ctDNA

Analysis of ctDNA has proven to be a minimally invasive diagnostic tool for people with cancer. For OC especially, ctDNA has repeatedly been shown to have better diagnostic performance than the traditional biomarker CA-125 [13,28]. In a recent study, for example, ctDNA analysis showed higher sensitivity and specificity for recurrence than CA-125 and preceded radiological findings by an average of 10 months [28].

In this current study, we identified nine studies with significantly higher positivity rates and/or higher levels of ctDNA than in non-malignant individuals, with correspondingly high sensitivity and specificity (Table 1 and Table 2). In these studies, quantitative analysis of ctDNA was reported to have a relatively high specificity (range: 84–100%) and sensitivity (74–100%) for the diagnosis of OC, accompanied by a variable detection rate (14.47–100%) (Table 1 and Table 2).

It has been shown that patients with high ctDNA levels at baseline (without treatment) have a higher tumour burden (in terms of metastatic lesions) (*p* < 0.05) compared with patients with lower ctDNA levels [22]. Also, when comparing ctDNA detection levels and tumour stage, several studies have consistently shown that ctDNA mutation copy numbers were significantly higher in late stages (III–IV) than in early stages (I–II) [16,19,20,39,40,43,44]. We hypothesise that ctDNA could help to further define patients in terms of their prognosis.

As for methylation of promoter regions of cfDNA, it has been reported to have significant diagnostic potential [48,52,53,55,56]. These studies have emphasised the feasibility of detecting hypermethylation within the promoters of OC-specific genes (*HOXA9*, *HIC1*, *RASSF1A*, *DAPK1*, *SOX1*, *hTERT*, *ZNF154*, *C2CD4D*, *WNT6*, *SPARC*, and *SFRP1*) from plasma/serum samples for the diagnosis of OC patients. These studies have shown the high diagnostic sensitivity and specificity of these hypermethylated genes in OC patients compared with healthy participants, highlighting their diagnostic capabilities. More importantly, the methylation status of ctDNA in the blood of OC patients could be useful for early detection [58,59] and enable personalised treatment. At ASCO 2019, Liu et al. reported on GRAIL based on the prototype methylation technology that can detect the tissue of origin of several tumours with high accuracy (clinical trial NCT02889978). In the subsequent year (2020), cfDNA methylation analysis sponsored by GRAIL reported the potential of early disease detection (stage I and II) in >50 cancer types, including OC [60]. Given that epigenetic modification of promoter regions of tumour suppressor genes have been shown to be significantly methylated in both early (I and II) and advanced stages (III and IV) of OC [58,59], targeting these modifications could be used as a marker for the detection OC at an early stage.

Although a significant difference(*p* < 0.05) has been shown in patients having advanced disease (III–IV) with higher serum CA-125 than the early stages (I–II), a comparison of methylation patterns in both early and advanced stages showed no significant difference (*p* > 0.05) [52,53,54,55]. However, there was a significant association (*p* < 0.05) with abnormal methylation of tumour suppressor genes when comparing both early and advanced disease stages compared with normal healthy controls [48,52,53,55,56]. These reports suggest that aberrant gene promoter methylation is an early event in the development of OC and that its sensitivity in detecting early stages (I–II) of cancer emphasises the need to combine this biomarker for screening purposes to improve early detection of the disease, particularly in high-risk women.

Regarding concordance, some studies showed an overall moderate (62.5%) [20] to low (28%) concordance [18], while others reported a relatively high (>80%) concordance [15,29] between tissue biopsy and ctDNA. The lower concordance could be due to the fact that ctDNA captures the heterogeneity of the metastatic/primary tumour in patients compared with tissue biopsy, which only captures a small part of the primary tumour or metastatic lesion. Similarly, when comparing the tissue-based concordance of mutations in three different anatomical regions, namely the adnexa, intra-abdominal sites, and the peritoneal cavity, a high concordance was found for all three anatomical regions (81–89.6%) [15]. The similar concordances suggest that comparable amounts of cell-free mutant DNA are released from different tissues, demonstrating that liquid biopsies can be obtained from different anatomical sites such as ascitic fluid [61] for the efficient detection of residual occult disease in OC in the few rare cases where blood ctDNA or CA-125 levels remain negative or normal, respectively, when relapse occurs.

Interestingly, some studies have highlighted the diagnostic potential of ctDNA analysis in helping with the early detection of tumours in OC patients based on promoter methylation of selected genes such as *HOXA9* and *HIC1* [53,55]. In contrast, other studies have shown no statistical significance [43,54]. Of note, a positive correlation was found between the age group and the increase in methylation in the promoter region of the analysed genes. This could be related to an ageing process that is independent of the pathological or health status of the subjects and therefore requires further investigation to fully elucidate its significance.

There is some evidence in the literature for the predictive value of ctDNA playing a role. In high-grade serous ovarian cancer (HGSOC) (III and IV), dynamic analysis of ctDNA for BRCA1/2 reverse mutations or mutations in BRCA1 and TP53BP1 could predict response to PARP inhibitor therapy [30]. CtDNA analysis has shown TP53 to be a biomarker for predicting response to first-line therapy [14] or for dynamic tracking of patient response to therapy [18] in HGSOC patients. An important multicentre analysis of the genome-wide profile of CNV using machine learning models has shown that these aberrations in cfDNA can predict response to platinum-based therapy and progression-free survival (PFS) in epithelial ovarian cancers (EOCs) [24].

### 2.2. Prognosis and Monitoring of Treatment Response Using ctDNA

Multiple studies have evaluated the utility of ctDNA as a prognostic biomarker in OC. These studies have also demonstrated that ctDNA dynamics correlate with response to adjuvant chemotherapy in OC and may predict treatment response or disease progression similar to [13] or earlier [16,28,35] than the conventional OC marker CA-125. Early identification of recurrence is crucial for guiding treatment choice and shorter patient turn-around times, ultimately enhancing favourable clinical outcomes.

In monitoring OC patients undergoing upfront debulking surgery, the OS of these patients with higher mutant copies of ctDNA (>10 mutant copies/mL) in plasma were shown to be significantly worse (*p* = 0.008) than in those with fewer copies (<10 mutant copies/mL) [20]. Other recent studies in OC have reported on pre-operative ctDNA detection dynamics and associated them with patients’ clinical outcomes [23,28,43]. For instance, Ogasawara et al. [43], in analysing *PIK3CA* and *KRAS* mutant copies via digital droplet PCR (ddPCR) in 308 OC patients, found pre-operative ctDNA detection to be an indicator of PFS and overall survival (OS) (*p* = 0.0001 and *p* = 0.017, respectively). Multivariate analysis revealed that ctDNA remained an independent risk factor for recurrence (*p* = 0.010). Amongst eight patients who were ctDNA positive at the time of initial diagnosis, seven (87.5%) remained ctDNA positive at the time of their first recurrence. Hence, pre-operative ctDNA measurement in OC patients’ plasma/serum, holds promise as a predictive biomarker for tumour prognosis. Similarly, when exploring evolutionary trajectories in OC patients by longitudinal analysis of ctDNA, Kutz et al. [14] identified evolutionary patterns of *TP53* variants that had an association with relapse seeding clones from the primary tumour. Likewise, *TP53* mutations were undetectable in cfDNA during treatment (platinum-based chemotherapy) but reappeared at disease progression, illustrating the promise of *TP53* as a biomarker for disease monitoring [37]. Thus, *TP53* mutations in ctDNA can be used as biomarkers for longitudinal monitoring of treatment response and can help predict recurrence of the tumour. Additionally, in terms of time to progression (TTP), EOC patients with detectable ctDNA in their last on-treatment plasma sample exhibited a significantly shorter TTP (HR = 5.63, *p* < 0.001) and OS (HR = 8.22, *p* < 0.001) [13]. Also, going a little step further, Lheureux et al. [22] demonstrated the feasibility of using mutant fragment size distribution for computing TTP and disease-free survival (DFS). The authors reported that, at baseline, plasma samples with significant differences in mutant fragment size distribution in detected ctDNA (mutation in *TP53*) from HGSOC patients, had shorter TTP (*p* = 0.001). This mutation in *TP53* also showed shorter DFS for patients with a significant difference (HR, 4.8 (1.8–12.5), *p* = 0.001) in a mutation-specific fragmentation profile compared with those with no difference in this fragmentation. Likewise, in OC patient cohorts who were predominantly (92%) platinum-resistant, the plasma genome altered fraction and mutation burden (*p* = 0.011 and *p* = 0.041, respectively) were analysed using low-coverage WGS and were both associated with PFS [32]. Lastly, in terms of cancer-specific methylation profiling, detection of *HOXA9* during treatment with a poly ADP ribose polymerase inhibitor (PARPi) (Veliparib) was associated with worse clinical outcomes (PFS and OS) in platinum-resistant *BRCA*-mutated OC. Specifically, after three treatment cycles of the PARPi, patients with detectable *HOXA9* ctDNA had a shorter PFS and OS (*p* < 0.0001 and *p* = 0.002, respectively) compared with patients with undetectable ctDNA. In these OC patients, those with detectable *HOXA9* positive ctDNA at baseline but subsequent undetectable levels had the most favourable clinical outcomes, followed by patients with undetectable ctDNA throughout. Homologous recombination repair (HRR) restoration may have occurred in some of these patients, potentially affecting the responsiveness of the PARPi. Hence, algorithms that can be used to evaluate HRR restoration and other resistant mechanisms that can aid in predicting responsiveness to PARPi during the course of treatment in OC ctDNA [21], as discussed later in the next section, would have been evaluated in this study.

### 2.3. Identifying Therapeutic Resistance Using ctDNA

Treatment resistance in OC is a frequently observed phenomenon that is one of the main causes of treatment failure and poor patient outcomes [62]. Treatment with platinum-based chemotherapies or targeted therapies can lead to the emergence of resistant subclones with additional amplification or reversion of previous genetic aberrations. Others may be pre-existing resistant subclones [63]. Therefore, it is crucial to identify and monitor these mutated genes within the resistant subclone by ctDNA analysis.

Recently, neoadjuvant chemotherapy (NAC), i.e., a short course of chemotherapy of usually three cycles before interval debulking surgery, has been increasingly used as a primary treatment method instead of primary surgery followed by adjuvant chemotherapy [64]. In the context of NAC treatment, it has been repeatedly reported that NAC-sensitive OC patients have fewer genetic aberrations in the ctDNA compared with NAC-resistant patients before treatment [31,40]. NAC-resistant patients may not have achieved optimal debulking surgery compared with NAC-sensitive individuals, demonstrating yet another significant clinical utility of ctDNA in the NAC setting. Hence, a reduced tumour burden in the NAC-sensitive individuals may have accounted for the reduced ctDNA levels compared with the resistant counterparts. For instance, Sharbatoghli et al. [31], who investigated the copy number alteration (CNA) profile in HGSOC patients treated with NAC (carboplatin + paclitaxel), reported genes with CNAs that were only detected in the NAC-resistant group. These include *HSF1*, *MROH1*, *TMEM249*, *GSTT2B*, *ABR*, and *NOMO2*. Similarly, another previous report [40] has highlighted that EGFR gene amplification, along with mutations in the *SLITRK5*, *RET*, *NYAP2*, *FAT1*, *LRRTM1*, *BRINP2*, *GRM1*, *CDH9*, and *GRM1* genes, were only present in the ctDNA of post-NAC plasma samples in predominantly HGSOC patients [22]. Other novel pathogenic DNA mutations with biological mechanisms related to chemoresistance have also been recently reported [15] using ctDNA from HGSOC patients treated with NAC. Therefore, common mutant gene copies emerging or upregulated in these resistant subgroups of OC patients need to be carefully identified and their downstream mechanisms investigated to identify potential drug targets.

In addition, mutation profiles of genes in the pre-PARPi samples of *BRCA*-mutated OC patients were analysed. The results showed that a high tumour mutational burden and alterations in genes related to drug efflux and replication fork stabilisation are associated with PFS after PARPi. Subsequently, the authors observed an increased mutational burden after progression in the PARP inhibitors (89.7% of patients) and linked these resistance mechanisms to the restoration of HRR, replication fork stability, the upregulated survival pathway, loss of target, and drug efflux. The results showed poor PFS in patients with the restoration of HRR (*p* = 0.003) and in patients with concomitant involvement of two or more resistance mechanisms (*p* = 0.040) [21]. Similar results were previously reported [30] in OC ctDNA when pathogenic activatable mutations were analysed.

In a recent study [22] identifying emerging mechanisms of resistance to PARPi in HGSOC in a phase II clinical trial of cediranib plus Olaparib, the authors reported emerging mutations or aberrations of resistance to PARPi, including *BRCA1/2* reversion mutations, at baseline or during treatment, that may affect response to further therapy. CtDNA mutations in *ESR1* have been repeatedly reported to confer resistance in individuals with metastatic breast cancer [65]. The detection of significant levels of ctDNA mutant copies in HGSOC patients in a recent study [26] is a very important finding. The authors did not report the prognostic value in the patients who were positive for the mutated copies of *ESR1*. For this reason, the prognostic significance in a large and well-defined cohort of HGSOC patients is warranted. Also, in an earlier study [47], amplification of *ERBB2* was reported as a potential driver mutation conferring resistance to platinum-based chemotherapy in an OC patient. A ctDNA-guided therapy helped to select the second-line treatment of choice (trastuzumab), which improved the response to treatment and reduced the tumour burden.

### 2.4. Detection Techniques of ctDNA and Challenges in Ovarian Cancer

**Figure 1:**
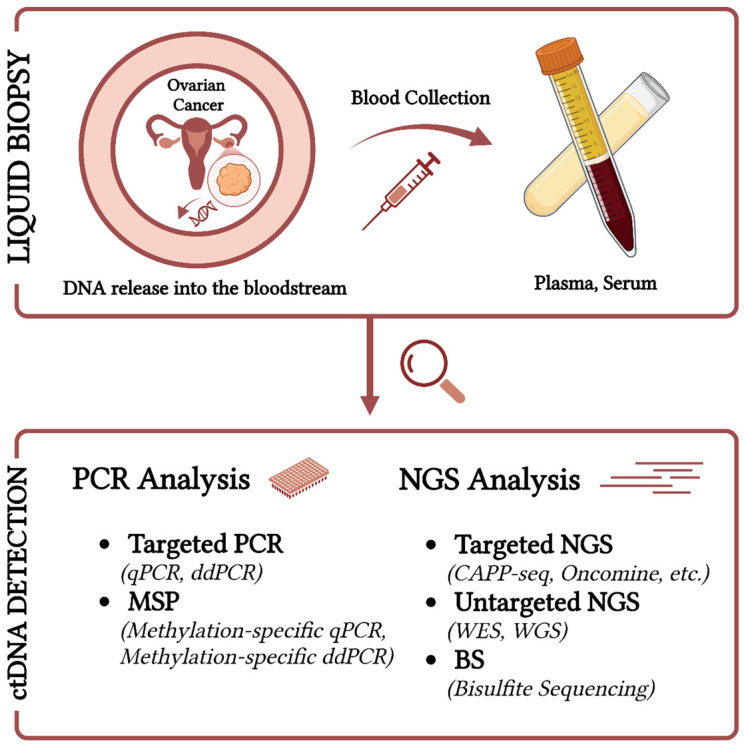
Detection methods of ctDNA in Ovarian Cancer. Created in https://BioRender.com (accessed on 30 December 2024).

Plasma/serum ctDNA analysis focuses on the development of new technologies for the effective quantification and characterisation of cancer-specific mutations in the peripheral blood of patients. Although the amount of tumour DNA has been shown to reflect the tumour burden, interpretation is often hampered by the presence of wild-type cfDNA from non-tumour cells. Therefore, a number of highly sensitive methods have been developed to detect aberrations in ctDNA, including mutation, amplification, chromosomal rearrangement, and hypermethylation [66] (Figure 1).

This challenge of detection is particularly due to the small amount of ctDNA in the enormous amount of wild-type cfDNA in the blood of cancer patients [67]. Therefore, sensitivity and specificity are crucial in detecting these cancer-related gene mutations in ctDNA.

Advances in next generation sequencing (NGS) and PCR protocols, together with an efficient bioinformatics pipeline, have enabled the quantitative detection of mutations with a low limit of detection (LOD) (≤0.001%) [66]. For example, in a recent study [13] on ctDNA in OC, the authors reported the use of ultrasensitive NGS assays capable of detecting ctDNA fractions down to 0.0004%.

In these current OC ctDNA studies, digital droplet PCR (ddPCR) quantifies ctDNA with higher sensitivity, efficiency, and precision, andmost importantly, it is easily reproducible [37]. However, prior knowledge of the target gene mutation is required. In contrast, NGS analysis, either by targeted or whole exome sequencing (WES)/genome sequencing (WGS), comprehensively interrogates multiple gene loci, allowing the identification of known driver genes and novel genetic alterations associated with the tumour [68].

The targeted queries selected genes, making them more specific but with lower gene coverage. In this current review, 20 studies used targeted NGS for the detection of ctDNA from plasma/serum, which serves as the most common detection platform [13,14,15,16,18,19,21,22,23,28,29,30,34,37,40,41,42,46,47]. Of critical note, the molecular barcoding technologies that are integrated into the targeted NGS approach used in some of the OC ctDNA studies, such as personalised cancer profile sequencing (CAPP-Seq) 4 [39,40] and Oncomine [18,37], help to improve sensitivity by reducing background sequencing errors.

The untargeted NGS approach (WES and WGS) has the advantage of being able to identify newly occurring aberrations during the course of treatment. However, it has the disadvantage of being relatively expensive and less sensitive [66,67,68]. Ten of the studies used WGS/WES [17,24,25,27,31,32,33,35,36,44].

Among the detected somatic mutations, the mutated *TP53* is still the most frequently identified gene in OC ctDNA analysis with high mutation copy numbers [15,18,20,25,32,37,39,40,47]. CtDNA studies in OC mostly focus on patients with HGSOC (Table 1) because it is the most common and aggressive subtype of EOC [69]. Molecular analyses in HGSOC tumours have shown a high prevalence (90%) of the somatic TP53 mutations subtype [70], making it an important target gene.

In this current review, the *TP53* mutant ctDNA was the most frequently mutated gene. Other frequently mutated genes include *BRCA1/2*, *KRAS*, *PIK3CA*, etc. (Table 1). Some studies [17,23,24,27,31,32,33,35,36,44] also investigated somatic copy number alterations (CNAs) as alternative targets for the detection of OC ctDNA. The detection of somatic CNAs in OC is important, especially in HGSOC, which is characterised by chromosomal instability and a high burden of CNAs [70].

Unique cancer methylation patterns at promoter sites can be detected in the ctDNA of OC patients (Table 2). Techniques used include methylation-specific PCR (MSP) [49,50,53,54,55,56,57], bisulfite sequencing [14], and genome-wide DNA methylation profiling [51] (Table 2).

MSP was the most important detection method for the detection of methylated ctDNA in the serum of OC patients. Sensitivity and specificity varied depending on the gene analysed (Table 2). In particular, *HOXA9* was the most frequently (5/10) identified gene marker. A larger sample size is required to assess its prognostic significance.

Regarding the analytes used, more plasma samples (42/45) than serum (3/45) were used for ctDNA analysis (Table 1 and Table 2). Nevertheless, particularly for cfDNA methylation profiling, where serum was predominantly used as the predominant analyte for DNA extraction in an earlier OC ctDNA review [11], this time, plasma (8/10) was the predominant analyte compared with serum (2/10) (Table 2). This paradigm shift in the use of plasma as an analyte for cfDNA extraction and for subsequent ctDNA analysis may be due to the significantly higher ctDNA detection in plasma compared with serum from paired samples [71]. A very important pre-analytical step that is often overlooked is cfDNA extraction. As the mutant copies are sparse in the enormous amount of wild-type DNA, inefficient extraction protocols might miss ctDNA in the plasma/serum samples used. None of the studies used new optimisation procedures, as previously described [72,73], to increase the yield of ctDNA. Hence, optimisation protocols that can help increase cfDNA yield are warranted in OC ctDNA studies, especially during longitudinal tracking of this biomarker in patients during clinical trials.

Overall, there appear to be new and improved techniques for the identification of mutant copies of ctDNA using NGS platforms, which has greatly increased sensitivity and specificity. In contrast, the identification of methylated gene panels appears to be less sensitive and specific compared with previous studies in OC ctDNA [11]. Therefore, methylation analysis needs to be improved to increase sensitivity and specificity.

## 3. Discussion

Due to rapidly developing high-throughput technologies, liquid biopsy is expected to become an essential part of the tools for the clinical treatment of cancer patients in terms of precision medicine. The scientific community is working hard to show that liquid biopsy can provide dynamic tests that help physicians offer the best treatment. In this context, ctDNA, one of the targets of liquid biopsy, has attracted particular interest as it has been shown to be a potential biomarker for diagnosis, prognosis, and therapy in many types of cancer [74].

Patient numbers in current studies (Table 1 and Table 2) have significantly improved compared with earlier studies [11,74] before 2020. Recruitment of participants into large prospective studies or clinical trials should be widely performed and encouraged as this will aid in increasing the statistical power and help draw meaningful conclusions in ctDNA studies in OC patients.

Secondly, plasma—as the preferred analyte for ctDNA analysis—should be leveraged and accepted across different platforms, potentially contributing to ctDNA analysis standardisation in studies using participants with OC. Hence, the standardisation of pre-analytical techniques and sample preparation is crucial for acceptance into routine clinical practise in the near future. More importantly, measures to strengthen this standardisation process are long overdue.

In addition, various methods/techniques have been used for the detection and quantification of ctDNA with relatively high diagnostic sensitivity and specificity, both of which are associated with high detection rates. However, only two studies [18,37] have used different detection platforms for the identification of ctDNA samples to validate the results obtained. All in all, the use of different platforms to confirm ctDNA results in OC studies is key to introducing them into routine clinical practise.

The TP53 detection rate in the ctDNA of HGSOC patients was also described as relatively high in this current review [18,37,42,47]. Other mutations identified in HGSOC, such as KRAS, PIK3CA, BRCA1/2, etc. (Table 1), should not be overlooked. Similarly, gene mutations, such as PIK3CA, KRAS, PTEN, APC, RGFR, ARID1A, etc., were more common in other EOCs (non-HGSOC) such as clear cell, endometrioid, mucinous, and low-grade serous EOCs [19,20,39,43]. Of those who analysed ctDNA using different OC subtypes, some authors did not report the association with gene mutations. Therefore, such analysis is warranted in future studies as this information may aid in treatment decisions and ultimately disease management.

Furthermore, plasma-based tests for ctDNA can provide invaluable information about the status of a patient’s cancer. However, there are numerous alternative sources of ctDNA that may offer unique advantages in certain settings. In OC, non-blood sources of ctDNA include peritoneal fluid and ascites [75]. More importantly, ascites is more commonly detected at diagnosis and at relapse in patients with advanced-stage OC than in any other cancer [76]. Ascites has been reported to yield large amounts of tumour cfDNA and could be an alternative to tumour sampling for BRCA testing [61]. Several studies [15,29,42] comparing OC ctDNA and tumoural/mutant cfDNA from ascites of comparable patient samples have shown good concordance and feasibility of using ascites to map the mutational landscape of the tumour. Therefore, the analysis of cfDNA from ascites together with its circulating counterpart, i.e., ctDNA, could be approved and implemented, especially in the advanced stages of the disease.

More recently, liquid biopsy analyses have focused on cfDNA fragmentation and have helped in the assessment of fragmentomic markers such as fragment sizes, preferred ends, end motifs, and nucleosomal footprints of normal and cancer cells from individuals [77].

In OC ctDNA analysis, recent studies [17,22,25,51] have shown that analysing ctDNA fragmentation is a crucial diagnostic and prognostic tool.

One study [22] using fragmentomics went a small step further and included clonal haematopoiesis of indeterminate potential (CHIP) in its ctDNA analysis. CHIP was identified in some patients with new TP53 mutations. This means that cfDNA fragmentation profiling can distinguish potential CHIP mutations from tumourigenic variants in the absence of paired peripheral blood mononuclear cells [22]. This will help to identify “true” mutant copies from tumours compared with CHIP-induced variants from normal cells, especially in the elderly. However, large prospective studies are needed to validate this technological advancement in OC patients.

Finally, new and emerging bioinformatics tools based on artificial intelligence (AI) have recently emerged for ctDNA analysis [78]. AI-based variant calling helps to reduce the cost, difficulty, and complexity associated with NGS protocols and can provide robust results even with very low sample volumes [78,79,80]. In this current review, a ctDNA-based AI study [51] showed diagnostic potential with high specificity (>80%) and sensitivity (100%). However, the clinical applicability with regard to the prognosis and longitudinal disease monitoring of the disease must be evaluated in clinical studies.

## 4. Conclusions

**Figure 2:**
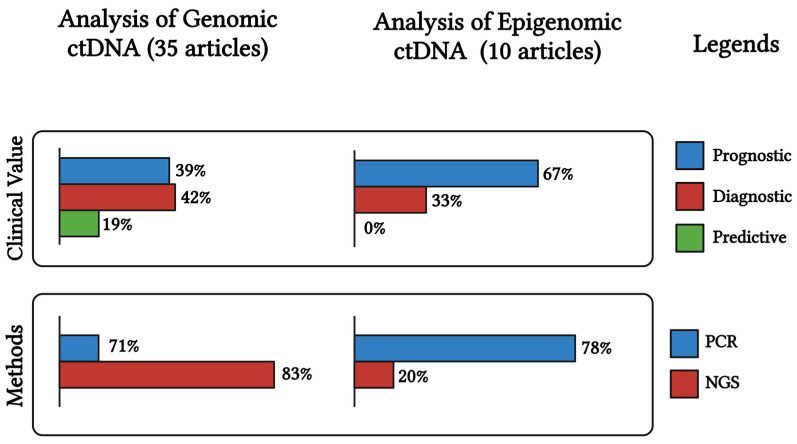
Genomic and epigenomic ctDNA analysis in ovarian cancer. Created in https://BioRender.com (accessed on 30 December 2024).

Overall, the evaluation of ctDNA in blood samples taken before or after surgical or chemotherapeutic treatment could enable effective monitoring of patients and detection of residual occult disease, facilitating the early detection of disease progression and the tailoring of adjuvant therapies for OC treatment. Surveillance with ctDNA as a liquid biopsy could aid in the early detection of resistance in patients undergoing therapy and help to intercept these actionable genetic vulnerabilities, ultimately supporting the selection of a more rational second-line treatment. As mentioned previously (Section 2.1), ctDNA analysis can anticipate disease symptoms about 10 months earlier. The occurrence of genetic mutations at recurrence means that ctDNA can accurately represent the heterogeneity of malignant lesions and can be very helpful in monitoring the disease progression. According to current knowledge, both genomic and epigenomic ctDNA analyses can be useful for the clinical management of OC. However, genomic ctDNA analysis seems promising for its approval into clinical practise as numerous studies have been performed and more than 2800 OC patients have been analysed. Genomic ctDNA analysis is potentially suitable for diagnosis, prognosis, and predictive medicine in OC (Figure 2). It is noteworthy that the majority of studies have utilised NGS technologies. In contrast, epigenetic studies of ctDNA require further investigation in order to better understand the potential clinical utility of methylation profile differences in OC patients.

All in all, ctDNA is a promising tool for assessing the tumour burden in OC patients. In addition, large prospective studies are needed to determine the clinical utility of ctDNA detection for the early diagnosis of OC and its impact on patient outcomes. However, we can assume that both genomic and epigenetic ctDNA analyses could become interesting for diagnosis, follow-up, and therapy in the coming years due to advances in high-throughput technologies and the potential of AI to create a personalised profile of the OC patient for clinical management.

## Figures and Tables

**Table 1:** Studies on ctDNA and in Ovarian Cancer: 2019–2024.

OC Subtype & Stage	Patients and Controls	Age (yrs)	Plasma or Serum	Biomarker	Detection Method	Detection Rate (%)	Sensitivity and Specificity	Clinical Value	Refs
EOC/I–IV	P= 63	30–80	Plasma	21 gene panel, including *TP53*, *CLDN19*, *ARID4B*, etc.	NGS	Diagnosis = 93 Progression = 100	NR	Prognostic: OS: HR = 6.60, *p* < 0.001 TTP: HR = 4.69, *p* < 0.001	(Kallio et al., 2024) [13]
High- and low-grade serous EOC/I–IV	P = 15 (Low grade = 1; High grade = 14)	47–82	Plasma	275 cancer-associated genes, including *TP53*, *KMT2A*, *NOTCH1*, *KDM5C*, *ARID1B*, etc.	NGS	100	-	Diagnostic/Predictive/Longitudinal monitoring	(Kutz et al., 2024) [14]
HGSOC/II–IV	P = 29 (Platinum resistant = 14; Platinum sensitive = 15)	Platinum resistant = 57–80 Platinum sensitive = 59–81	Plasma	700 cancer-associated genes, including *TP53*, *KCNH2*, *JAK2*, *GRIN2A*, *FGFR3*, *ARID1B*, etc.	NGS	Pre-treatment = 82.8, Relapse = 89.5	NR	Prognostic: PFS: R = −0.72, *p* = 0.008 OS: R = −0.74, *p* = 0.005	(Marchi et al., 2024) [15]
EOC/I–IV	P = 296 B = 95	58.1 ± 12.3 (median)	Plasma	9 gene panel, including *TP53*, *BRCA1/2*, *PTEN*, etc.	NGS	89.9	Sn = 92% Sp = 84%	Prognostic: PFS: HR = 10.71, 95% CI: 4.43–25.9	(Heo et al., 2024) [16]
EOC/I–IV	P = 591 HC = 204 B = 253	P = 37–85 HC = 47–75 B = 19–92	Plasma	cfDNA fragmentomes	WGS	NR	Sn = 72%, 69%, 87%, 100% Sp > 99%	Diagnostic	(Medina et al., 2024) [17]
HGSOC/III–IV	P = 10	NR	Plasma	10 gene panel, including *TP53*, *AKT1*, *KRAS*, *PIK3CA EGFR*, etc.	Two NGS platforms plus ddPCR	Accel = 60 Oncomine = 100	Oncomine: Sn ≥ 90%, Sp ≥ 99% Accel: Sn = 90% Sp ≥ 99%	Longitudinal monitoring/Predictive	(Calapre et al., 2023) [18]
EOC/I–IV	P = 29	18–85	Plasma	50 gene panel, including *TP53*, *KRAS*, *PIK3CA*, etc.	NGS	82.8	NR	Prognostic: OS: HR = 6.56, 95% CI: 1.07–40.17	(Chao et al., 2023) [19]
EOC/I–IV	P = 41 B = 6 BOT = 9	NR	Plasma	Mutant genes, including *TP53*, *KRAS*, *PIK3CA*, etc.	dPCR	58	NR	Prognostic: Log-rank test: OS (*p* = 0.008)	(Dobilas et al., 2023) [20]
EOC/I–IV	P = 54	43–79	Plasma	531 gene panel, including *TP53*, *BRCA1/2*, *CHEK2*, etc.	NGS	89.7	NR	Prognostic: PFS (*p* = 0.0003)	(Kim et al., 2023) [21]
HGSOC	P = 30	63 (median)	Plasma	*BRCA1/2*, *TP53*, *TP53*, *PALB2*, *CCNE1*, etc.	NGS	74.4	NR	Prognostic: DFS: HR = 4.79, 95% CI: 1.84–12.5	(Lheureux et al., 2023) [22]
HGSOC, LGSOC/I–III	P = 48	58.9 (median)	Plasma	59 gene panel, including *BRCA1/2*, *TP53*, *BRAF*, *RB1*, etc.	NGS	77.4	NR	Prognostic: RFS: HR = 2.06, 95% CI: 0.60–7.10, *p* = 0.23	(Zhu et al., 2023) [23]
EOC/II–IV	P = 44 HC = 17	NR	Plasma	CNV	LC-WGS	NR	NR	Diagnostic/Predictive	(Chen et al., 2023) [24]
EOC/I–IV	P = 59 HC = 100	27–82	Plasma	OC score (CNV, nucleosome footprint, 5′ end motifs, fragmentation profiles)	Low-pass WGS	I–II = 85.7	Sn = 97.7% Sp = 94.7%	Diagnostic	(Zhou et al., 2023) [25]
HGSOC/I–IV	P = 80 HC = 11	<39 and ≥41 (median)	Plasma	*ESR1* mutations	ddPCR	13.8	NR	Diagnostic	(Stergiopoulou et al., 2022) [26]
EOC and non-EOC/I–IV	P = 271 B = 130 BOT = 41 Invasive = 92 Metastatic = 8	B = 43–64, BOT = 37–63, Invasive = 57–73, Metastatic = 52–69	Plasma	Nucleosome footprint and CNA	LC-WGS	NR	Nucleosome footprint: AUC = 0.71 (95% CI: 0.65–0.77) CNA: AUC = 0.72 (95% CI: 0.66–0.78)	Diagnostic	(Vanderstichele et al., 2022) [27]
EOC/I–IV	P = 69 (Cohort A, pre-surgical = 44; Cohort B, post-surgical and/or after therapy = 12; Cohort C, after completed treatments = 13)	29–82 (55.5 median)	Plasma	*TP53*, *ARID1A*, *KRAS*, etc.	Tumour-informed multiplex PCR NGS	Cohort A = 73 Cohort B = 33 Cohort B and C = 23	Sn = 100 Sp = 100	Prognostic: RFS: HR = 7.34, 95% CI: 0.75–72.3, *p* = 0.087	(Hou et al., 2022) [28]
EOC/II–IV	P = 18 (6 paired ascites and 8 paired tumour tissues)	24–70	Plasma	333 cancer-related genes, including *TGFBR2*, *ARID1A*, *ATR*, *BCR*, *KMT2C*, etc.	NGS	94.4	NR	Diagnostic/Predictive	(Jie et al., 2022) [29]
HGSOC/III–IV	P = 18	48–79	Plasma	65 cancer-related genes, including *TP53*, *BRCA1/2*, *POLE*, *MSH3*, *ATR*, etc.	NGS	NR	NR	Diagnostic/Predictive	(Paracchini et al., 2022) [30]
EOC/II–IV	P = 6	38–78	Plasma	CNA	WGS	NR	NR	Prognostic: OS (*p* < 0.0001)	(Sharbatoghli et al., 2022) [31]
HGSOC, LGSOC, Clear cell, endometrioid, carcinosarcoma	P = 24	21–71	Plasma	Identified CNAs and mutations in other cancer-related genes such as *TP53*	WGS, WES	88	NR	Prognostic: PFS (TMB: HR = 8.6, 95% CI: 1.4–52; GAF: HR = 8.9, 95% CI: 0.91–87)	(Sabatier et al., 2022) [32]
EOC/III–IV	P = 109 (Upfront group = 23; CTX group = 9; Follow-up group = 13)	22–84	Plasma	CNV (CNI scores)	WGS	Chemo naive = 78, Platinum-eligible recurrent = 83.3, Non-platinum-eligible recurrent = 82.6	Sn: primary and recurrent = 87%, primary only = 78–91% Sp = 95–100%	Diagnostic	(Braicu et al., 2021) [33]
SOC/I, III, and IV	P = 138	31–81	Plasma	150 cancer gene panel, including *TP53*, *KRAS*, *LRP1B*, *ZNF703*, *NF1*, etc.	Hybrid capture-based NGS	83	NR	Diagnostic	(Shen et al., 2021) [34]
HGSOC/III–IV	P = 46	21–81	Plasma	CNA	Shallow WGS	14.47	NR	Prognostic: PFS: HR = 3.31, 95% CI: 1.33–9.13, *p* = 0.011	(Paracchini et al., 2021) [35]
EOC and non-EOC/I–IV	P = 80 (Malignant = 58 B = 66; BOT = 10) HC = 82	Malignant = 12–77, B = 21–82, BOT = 27–62, HC = 22–36	Plasma	CNA	WGS	NR	NR	Diagnostic	(Zhang et al., 2021) [36]
HGSOC/III–IV	P = 20	37–75	Serum	Total of 51 genes: 41 for Ampliseq and 10 for Oncomine, including *TP53*, *PIK3CA*, *ESR1*, etc.	NGS, ddPCR	85	NR	Diagnostic/Predictive	(Vitale et al., 2020) [37]
EOCs/III–IV	P = 39	NR	Plasma	ALU	qPCR	NR	NR	Diagnostic	(Waki et al., 2020) [38]
EOC/I–IV	P = 51	28–82	Plasma	197 cancer-related genes, including *TP53*, *APC*, *KRAS*, *EGFR*, *MET*, *PIK3CA*, etc.	Deep NGS (CAPP-Seq)	94	NR	Prognostic: PFS (*p* = 0.048)	(Noguchi, Iwahashi, et al., 2020) [39]
HGSOC and mucinous/III–IV	P = 10	44–74	Plasma	197 cancer-related genes, including *TP53*, *APC*, *KRAS*, *EGFR*, *MET*, *PIK3CA*, etc.	Deep NGS (CAPP-Seq)	100	NR	Diagnostic	(Noguchi, Sakai, et al., 2020) [40]
HGSOC/III–IV	P = 7	54–78	Plasma	26 genes, including *TP53*, *APC*, *KRAS*, *PTEN*, *PIK3CA*, etc.	NGS	86	NR	Diagnostic/Predictive	(Jagelkova et al., 2020) [41]
HGSOC/III–IV	P = 10	44–65	Plasma	88 genes, including *TP53*, *PIK3CA*, *MYC*, etc.	NGS	60	NR	Diagnostic	(Han et al., 2020) [42]
EOC/I–IV	P = 306	33–80	Plasma	2 genes: *KRAS* and *PIK3CA*	ddPCR	27.1	NR	Prognostic: PFS (*p* = 0.0001) OS (*p* = 0.017)	(Ogasawara et al., 2020) [43]
High- and low-grade serous EOC/I–IV	P = 70	NR	Plasma	CNV	WGS	Stage (I and II) = 55.56 Stage (III and IV) = 85.71	NR	Diagnostic/Predictive	(Wang et al., 2020) [44]
EOC/I–IV	P = 37 HC = 28	HC = 30–63 OC = 37–80	Plasma	*ALU*, *LINE 1*	qRT-PCR	NR	NR	Diagnostic	(Stamenkovic et al., 2020) [45]
EOCs (96% HGSOC)	P = 112	33–82	Plasma	54 cancer-related genes, including *BRAC1*, *BRAC2*, *TP53*, etc.	NGS	*TP53* = 96	NR	Prognostic: PFS: HR = 0.12 (*p* < 0.0001)	(Lin et al., 2019) [46]
HGSOC/II–IV	P = 12	68 (median)	Plasma	CNV and >500 cancer-related genes, including *TP53*, *PTEN*, *BRCA2*, etc.	NGS	100 for *TP53* and variable for the other genes	NR	PFS (*p* < 0.01)	(Oikkonen et al., 2019) [47]

BOT: borderline; B: benign; CAPP-seq: cancer personalized profiling by deep sequencing; CNA: copy number alteration; CNI: copy number index; CNV: copy number variation; CR: chromosomal rearrangement; DFS: disease-free survival; ddPCR: droplet digital PCR; dPCR: droplet polymerase chain reaction; EOC: epithelial ovarian cancer; GAF: genome-altered fraction; HC: healthy controls; HGSOC: high-grade serous ovarian cancer; HR: hazard ratio; LC-WGS: low-coverage whole genome sequencing; NGS: next generation sequencing; NR: not reported; OS: overall survival; P: patients; PFS: progression-free survival; qPCR: quantitative PCR; RFS: recurrence-free Survival; SOC: serous ovarian cancer; Sn: sensitivity; Sp: specificity; TTP: time to progression; TMB: tumour mutational burden; WES: whole exome sequencing; WGS: whole genome sequencing.

**Table 2:** Studies on epigenetic modification of cfDNA in Ovarian Cancer: 2019–2024.

OC Subtype & Stage	Patient/ Controls	Age (yrs)	Plasma/ Serum	Biomarker	Detection Method	Detection Rate (%)	Sensitivity and Specificity	Clinical Value	Refs
EOC/I–IV	P = 70 B = 39 HC = 4	OC = 52–64, Controls = 48–66	Plasma	*ZNF154*, *C2CD4D*, and *WNT6*	Bisulphite sequencing	82.8	Sn = 80% Sp = 97.6%	Diagnostic	(Herzog et al., 2024) [48]
EOCs	P = 19	38–86	Plasma	*Alu115*, *IFFO1*, and *CDH5* promoters	Methylation-specific qPCR	*Alu115* = 100, *IFFO1Me* = 66, *CDH5UM* = 86	NR	Prognostic: PFI: HR = 3.21, 95% CI: 1.15–9.00, *p* = 0.008	(Werner, Sjoquist, et al., 2024) [49]
EOC/I–IV	P = 125 HC = 72	Non-malignant = 25–85, Cancer = 21–83	Plasma	miR-200c and miR-141 genes	Methylation-specific PCR	NR	NR	Prognostic: OS: HR = 1.53, 95% CI: 1.0–2.35, *p* = 0.049	(Gahlawat et al., 2023) [50]
EOC/II–III	P = 5 HC = 12	P = 66.2 ± 18.14; HC: 67.8 ± 12.96 (median)	Plasma	*ANO2*, *ATP11A*, *AGAP1*, *ARFGEF2*, *BBS9*, etc.	AI and GW-DMP	100	GW-DMP: Sn = 95%, Sp = 100%; AI: Sn = 100%, Sp = 88%	Diagnostic	(Bahado-Singh et al., 2022) [51]
EOC/I–IV	P = 79 HC = 64	25–86	Plasma	*HOXA9*	Methylation-specific ddPCR	59.5	Sn = 37.5–59.5% Sp = 95.3%	Diagnostic	(Faaborg et al., 2021) [52]
EOC/I–IV	P = 85	20–65	Serum	*RASSF1A*, *DAPK1*, *SOX1*, *HOXA9*, *HIC1*, *SPARC*, and *SFRP1*	Multiplex methylation-specific qPCR	82.3–61.3	Varies with each gene. Sn = 85.88–72.94% Sp = 88.57–77.14%	Diagnostic	(Singh et al., 2021) [53]
EOC/I–IV, platinum resistant	P = 32	46–70	Plasma	*HOXA9*	Methylation-specific ddPCR	62	NR	Prognostic: PFS (*p* < 0.0001) OS (*p* = 0.002)	(Rusan et al., 2020) [54]
EOC/I–IV	P = 44	18–70	Serum	*HOXA9* and *HLC1*	Multiplex methylation-specific qPCR	*HOXA9* = 62.2 *HLC1* = 71.1	Sn = 88.9% Sp = 100%	Diagnostic	(Singh et al., 2020) [55]
EOC/I–III	P = 17 B = 15 HC = 15 A = OC group (P) B = Control group (B and HC)	32–68	Plasma	*hTERT*	Methylation-specific PCR	Group A = 70.6 Group B = 20	Group A: Sn = 76.9% Sp = 50%; Group B: Sn = 50% Sp = 90.9%	Diagnostic	(Li et al., 2020) [56]
EOC/I, III, and IV, platinum resistant	P = 23	41–81	Plasma	*HOXA9*	Methylation-specific dPCR	NR	NR	Prognostic: OS (*p* = 0.01)	(Thomsen et al., 2019) [57]

AI: artificial intelligence; B: benign; ddPCR: droplet digital PCR; dPCR: droplet polymerase chain reaction; EOC: epithelial ovarian cancer; GW–DMP: genome-wide DNA methylation profiling; HC: healthy controls; HR: Hazard ration; NR: not reported; OS: overall survival; P: patients; PFI: paracentesis-free survival; PFS: progression-free survival; qPCR: quantitative PCR; Sn: sensitivity; Sp: specificity.
